# Neutrophil extracellular traps-triggered impaired autophagic flux via METTL3 underlies sepsis-associated acute lung injury

**DOI:** 10.1038/s41420-022-01166-3

**Published:** 2022-08-27

**Authors:** Mengdi Qu, Zhaoyuan Chen, Zhiyun Qiu, Ke Nan, Yanghanzhao Wang, Yuxin Shi, Yuwen Shao, Ziwen Zhong, Shuainan Zhu, Kefang Guo, Wankun Chen, Xihua Lu, Zhiping Wang, Hao Zhang, Changhong Miao

**Affiliations:** 1 Department of Anesthesiology, Zhongshan Hospital, Fudan University; Cancer Center, Zhongshan Hospital, Fudan University, Shanghai, China; 2 Shanghai Key laboratory of Perioperative Stress and Protection, Shanghai, China; 3 Department of Anesthesiology, Affiliated Cancer Hospital of Zhengzhou University, Henan Cancer Hospital, Zhengzhou, China; 4 Department of Anesthesiology, Affiliated Hospital of Xuzhou Medical University, Xuzhou, China; 5 Department of Anesthesiology, Jinshan Hospital, Fudan University, Shanghai, China

**Keywords:** Sepsis, Sepsis

## Abstract

Neutrophil extracellular traps (NETs) assist pathogen clearance, while excessive NETs formation is associated with exacerbated inflammatory responses and tissue injury in acute lung injury (ALI)/acute respiratory distress syndrome (ARDS). Autophagy is generally considered to be a protective process, but autophagy dysfunction is harmful. Whether and how NETs affect autophagic flux during sepsis-induced ALI are currently unknown. Here, we confirmed that the level of NETs was increased in ARDS patients and mice models, which led to impairment of autophagic flux and deterioration of the disease. Mechanistically, NETs activated METTL3 mediated m^6^A methylation of Sirt1 mRNA in alveolar epithelial cells, resulting in abnormal autophagy. These findings provide new insights into how NETs contribute to the development of sepsis-associated ALI/ARDS.

## Introduction

The acute lung injury (ALI)/acute respiratory distress syndrome (ARDS) is one of the most life-threatening forms of acute respiratory failure featured with diffuse alveolar damage secondary to pulmonary or systemic inflammation process [1]. This acute inflammatory lung injury results in hypoxemia, pulmonary edema and other clinical manifestations [2]. As the leading cause of death among patients with sepsis, ALI/ARDS accounts for 10% of intensive care unit (ICU) admissions with a high death rate ranging from 35% to 46% depending on the degree of lung injury severity [3, 4]. To date, supportive therapy with mechanical ventilation remains the cornerstone of management [5]. Given that treatment options for ARDS are limited, it is crucial to explore their underlying mechanisms to develop interventions in the future.

During sepsis, the innate immune system plays a vital role in systemic inflammation and immunosuppression [6]. Neutrophils are the main members of innate immune cells, uncontrolled activation and infiltration of neutrophils would place the host at risk and promote deterioration clinically [7]. Neutrophil extracellular traps (NETs) released from neutrophils are networks of extracellular fibril matrices containing DNA and histones, embedded with proteases, such as myeloperoxidase (MPO) and neutrophil elastase (NE) [8]. As a double-edged sword in infection, NETs contribute to antibacterial defense, while excessive NETs production could mediate tissue injury in various inflammatory diseases, including sepsis [9]. Increasing studies have demonstrated that NETs could exacerbate inflammatory damage and coagulation disorder in sepsis [10–12]. Therefore, prevention of sepsis-induced lung injury by inhibiting NETs may be effective.

Autophagy helps cells deal with excess or defective organelles, mainly including macroautophagy, microautophagy, and chaperone-mediated autophagy, and macroautophagy is the most typical [13]. Most studies support that autophagy is an adaptive and protective biological process, but excessive or defective autophagy are harmful [13]. Accumulating evidence suggest that autophagy regulation appears to be protective against multiple organ injuries in sepsis models [14–16], although other studies have found the opposite [17, 18]. Whether autophagy is protective or deleterious depends on different stages of disease, stimulation and cell type, and therefore needs further investigation.

So far, whether and how NETs affect autophagy during sepsis-associated ALI/ARDS have not been clarified. Our study demonstrated that NETs were significantly elevated in ARDS patients and sepsis-induced ALI (SI-ALI) mice models. NETs caused impaired autophagic flux in alveolar epithelial cells, and this process was associated with changes in methylation status of Sirt1 mRNA mediated by METTL3.

## Results

### NETs formation is enhanced in ARDS patients and SI-ALI mice models

**Fig. 1:**
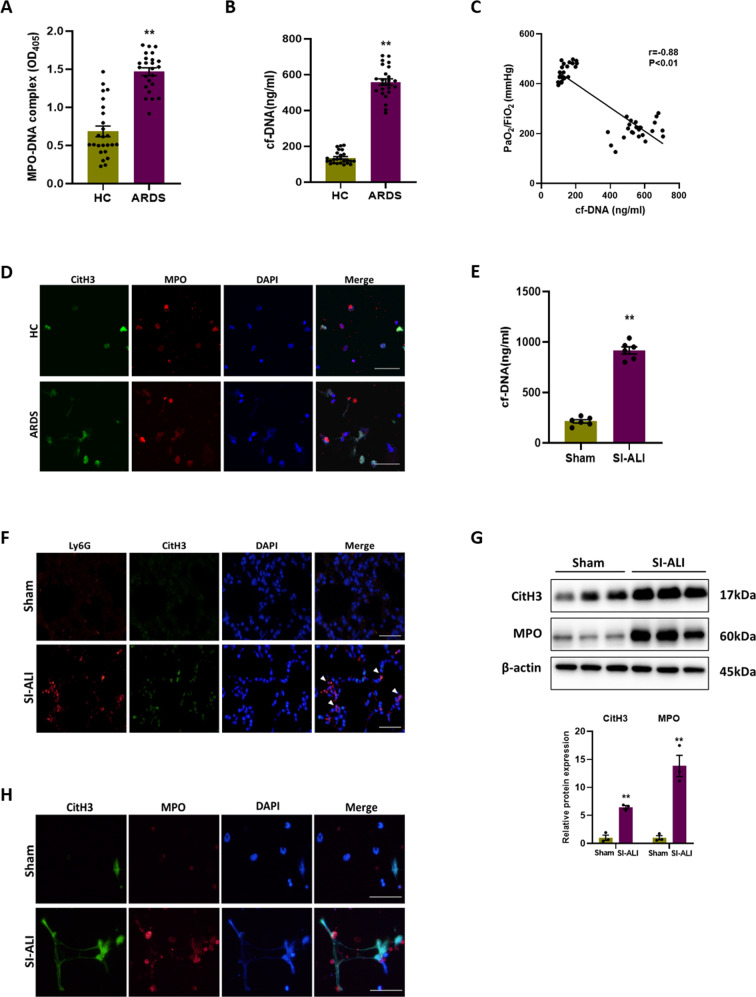
NETs formation is enhanced in ARDS patients and SI-ALI mice models. **A** MPO-DNA complex and (**B**) cf-DNA levels in serum of ARDS patient (*n* = 24) and healthy control (HC) (*n* = 25). **C** Correlation curve between the cf-DNA and PaO2/FiO2. **D** Representative immunofluorescence images of NETs released by neutrophils isolated from ARDS patient and HC. Scale bar: 30 µm. **E** cf-DNA levels in plasma of mice models (*n* = 6). **F** Representative images of immunofluorescence staining of Ly6G and CitH3 in lung tissues. Scale bar: 50 µm. **G** Western blot images of CitH3 and MPO expression in lung tissues. **H** Representative immunofluorescence images of NETs released by neutrophils isolated from mice models. Scale bar: 50 µm. Each bar shows means ± SEM. Data comparison between two groups was analyzed by unpaired *t*-test. *p < 0.05, **p < 0.01 versus the sham group.

First, NETs were verified to be involved in sepsis-associated ALI/ARDS. The levels of MPO-DNA complex (Fig. 1A) and cell-free DNA (cf-DNA) (Fig. 1B) in ARDS patients were significantly higher than those in healthy controls, and there is a strong correlation between the level of cf-DNA and PaO2/FiO2 (*r* = −0.88, *P* < 0.01) (Fig. 1C). Besides, the neutrophils from ARDS patients exhibited an increased capacity of NETs formation compared to those from healthy controls, indicated by co-expression of CitH3 and MPO after stimulated by PMA (Fig. 1D). The Baseline characteristics of all enrolled subjects are listed in Supplementary Table 1. Meanwhile, we constructed a model of SI-ALI in mice by cecal ligation and puncture (CLP) (S1A, B). Similarly, comparing the SI-ALI group with the sham group, the cf-DNA level in plasma was higher (Fig. 1E). Immunofluorescence and western blot images of lung tissues showed more neutrophil infiltration and NETs formation in SI-ALI group (Fig. 1F, G). Moreover, the ability of NETs formation of neutrophils was stronger in SI-ALI group (Fig. 1H). Overall, our results suggest that NETs formation is enhanced in patients and mice with sepsis-associated ALI/ARDS.

### NETosis inhibition, NETs depletion and degradation alleviate SI-ALI

**Fig. 2:**
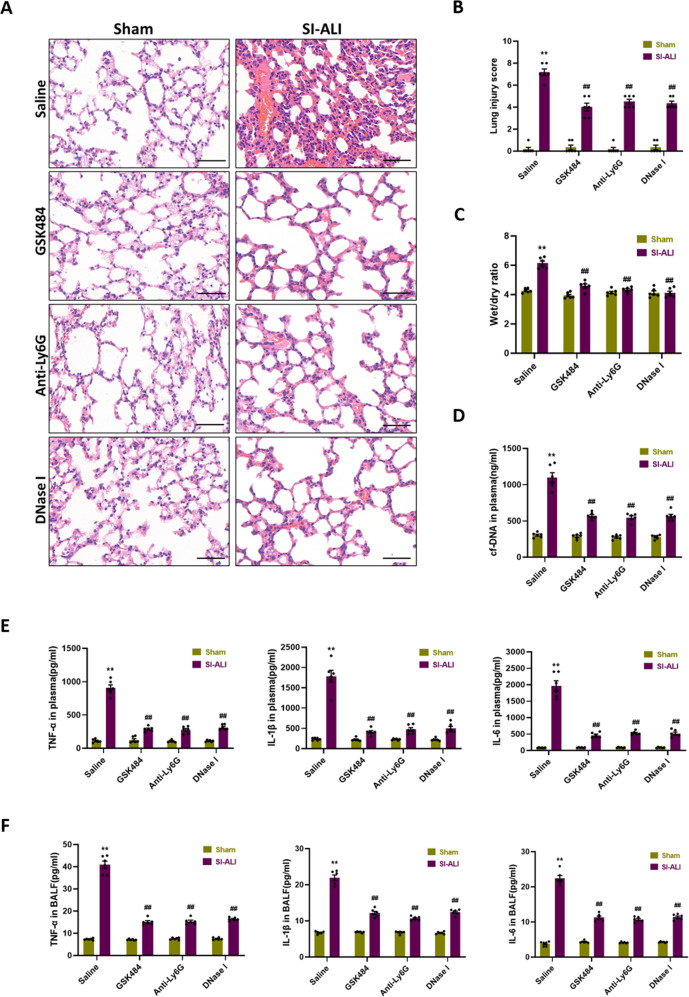
NETosis inhibition, NETs depletion and degradation alleviate SI-ALI. NETosis inhibition by PAD4 inhibitor, NETs depletion by anti-Ly6G and NETs degradation by DNase I protects against lung injury induced by CLP. **A** H&E staining of the lung tissues. Scale bar: 50 µm. **B** Semiquantitative histological scores of lung injury in groups (*n* = 6). **C** The lung wet/dry ratio (*n* = 6). **D** The levels of cf-DNA and (**E**) TNF-α, IL-1β, and IL-6 in plasma (*n* = 6). **F** The levels of TNF-α, IL-1β, and IL-6 in BALF (*n* = 6). Each bar shows means ± SEM. The comparison between two groups was performed using unpaired *t*-test. **p < 0.01 versus the sham group; ^##^*p* < 0.01 versus the saline group.

To determine if the formation of NETs in SI-ALI was pathogenic, mice were treated with peptidylarginine deiminase type 4 (PAD4) inhibitor (GSK484) to inhibit NETs formation, anti-Ly6G antibody to deplete neutrophils, and DNase I to degrade NETs respectively. H&E staining of mouse lung sections showed more serious haemorrhage and alveolar oedema, with thicker alveolar septa and more leukocyte infiltration following CLP for 24 h, while the administration of GSK484, anti-Ly6G antibody and DNase I significantly alleviated lung injury (Fig. 2A). Upon treatment, lung injury scores (Fig. 2B), pulmonary wet/dry ratio (Fig. 2C) and cf-DNA in plasma (Fig. 2D) all decreased. Furthermore, inflammatory cytokines (TNF-α, IL-1β and IL-6) in plasma and bronchoalveolar lavage fluid (BALF) were reduced, representing sepsis-induced inflammatory response was attenuated (Fig. 2E, F). These findings indicate that NETs contribute to SI-ALI and NETs-targeted therapies are protective.

### Autophagy is impaired in mice models of SI-ALI

**Fig. 3:**
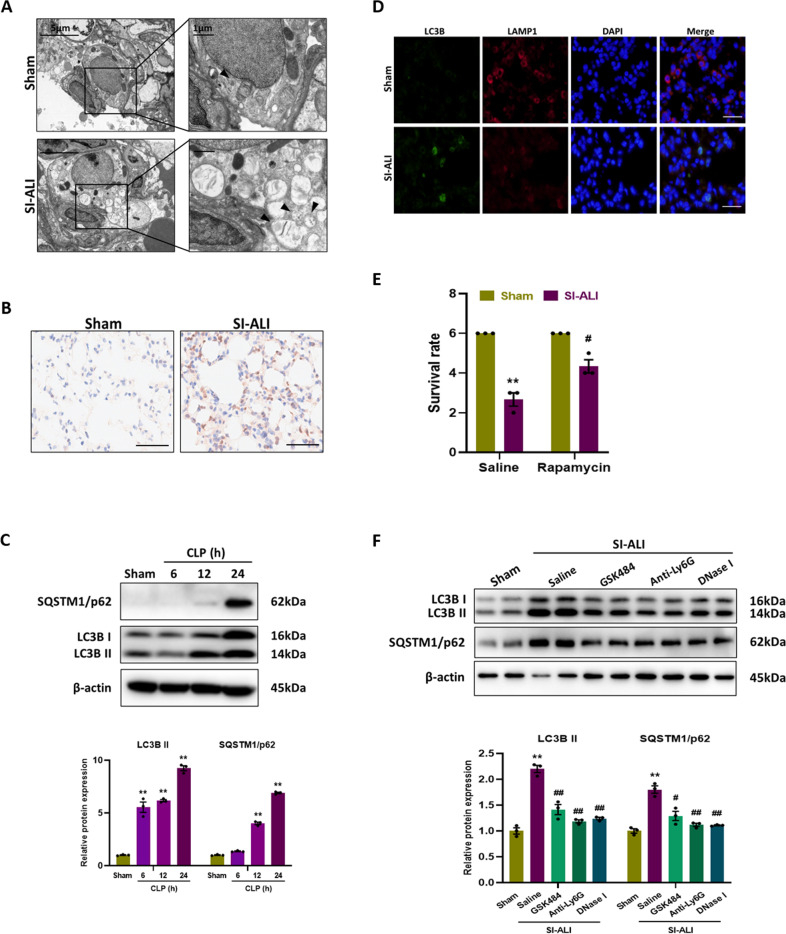
Autophagy is impaired in mice models of SI-ALI. Representative electron microscopic images of autophagic vesicles (black arrow) in lung tissues. **B** Representative images of immunohistochemical staining for LC3B in lung tissues. Scale bar: 50 µm. **C** Western blot images of autophagy signaling expression in lung tissues. **D** Representative images of immunofluorescence co-staining of LC3B and LAMP1 in lung tissues. Scale bar: 30 µm. **E** The survival rate of groups (*n* = 3). **F** Western blot images of autophagy signaling expression in lung tissues. Each bar shows means ± SEM. Data comparison between two groups was conducted by unpaired t-test. **p < 0.01 versus the sham group. ^#^*p* < 0.05, ^##^*p* < 0.01 versus the saline group.

Since the role of autophagy in septic lung injury is still unclear, we attempted to analyze the autophagy status in the CLP model in this study by observing autophagy vesicles in lung tissues with electron microscopy. Compared to sham group, there were more autophagic vesicles in SI-ALI group (Fig. 3A), with a higher level of LC3B (a hallmark of autophagy) showed by immunohistochemical images (Fig. 3B). Meanwhile, the expression of SQSTM1/p62, an autophagy substrate protein, was also detected. LC3B and SQSTM1/p62 protein levels were both elevated with time after CLP (Fig. 3C), and this simultaneous increase suggested that despite the enhancement in autophagy, autophagic flux was impaired. In addition, colocalization of lysosome (LAMP-1) and autophagosome (LC3B) was observed by immunofluorescence. The images showed SI-ALI group had a reduced LAMP-1 staining but an increased LC3B staining compared with sham group (Fig. 3D), indicating impaired autophagosome-lysosome fusion. All these results suggest that autophagy is impaired in mice models of SI-ALI.

We then treated mice with rapamycin, an autophagy activator, and found that rapamycin improved survival at 24 h after CLP (Fig. 3E), which further suggest that autophagy is dysfunctional. Rapamycin also alleviated lung injury, which was characterized by a decrease in lung injury scores (S2A, B), pulmonary wet/dry ratio (S2C), and inflammatory cytokines (TNF-α, IL-1β and IL-6) in plasma and BALF (S2D, E). What’s more, NETosis inhibition, NETs depletion and degradation could reduce the expression of SQSTM1/p62 (Fig. 3F), restoring the damaged autophagic flux. Taken together, the results suggest that NETs may be associated with impaired autophagic flux.

### NETs impair the cell viability via inducing autophagosome formation but blocking autophagic flux

**Fig. 4:**
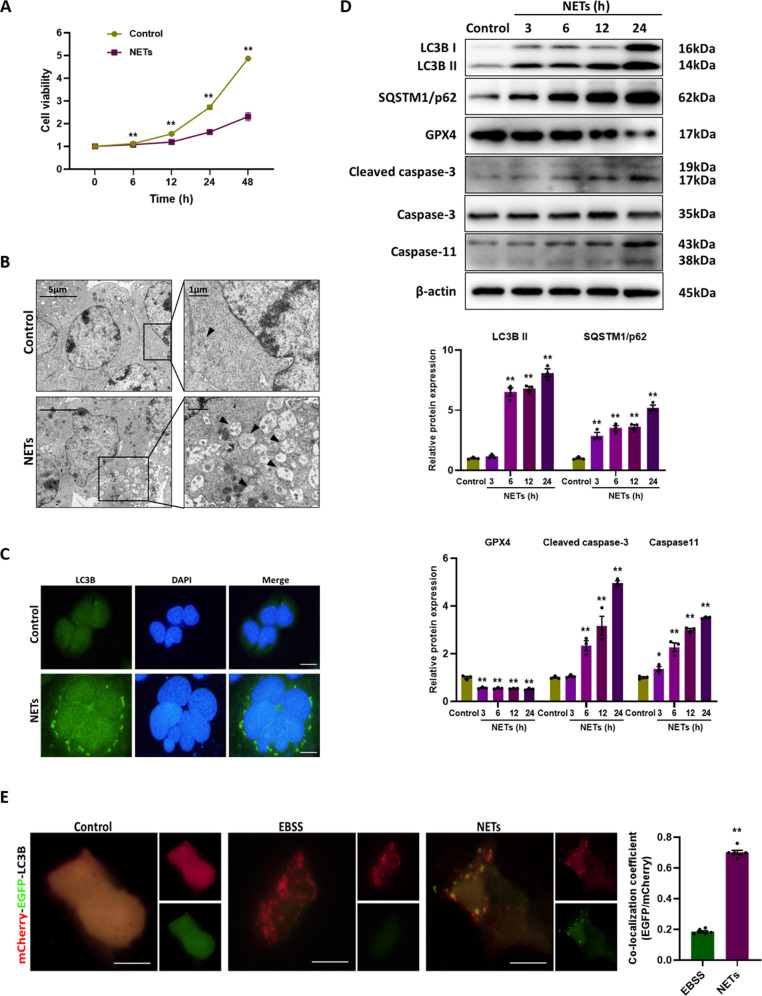
NETs impair the cell viability via inducing autophagosome formation but blocking autophagic flux. **A** The cell viability of alveolar epithelial cells with different treatments. **B** Representative electron microscopic images of autophagic vesicles (black arrow) in alveolar epithelial cells with different treatments. **C** Representative images of immunofluorescence staining of LC3B in alveolar epithelial cells with different treatments. Scale bar: 20 µm. **D** Western blot images of autophagy signaling and cell death markers expression in alveolar epithelial cells. **E** Representative fluorescent images of alveolar epithelial cells transfected with mCherry-EGFP-LC3B and treated with EBSS or NETs. Scale bar: 10 µm. Each bar shows means ± SEM. The comparison between two groups was conducted by unpaired *t*-test. *p < 0.05, **p < 0.01 versus the control group.

**Fig. 5:**
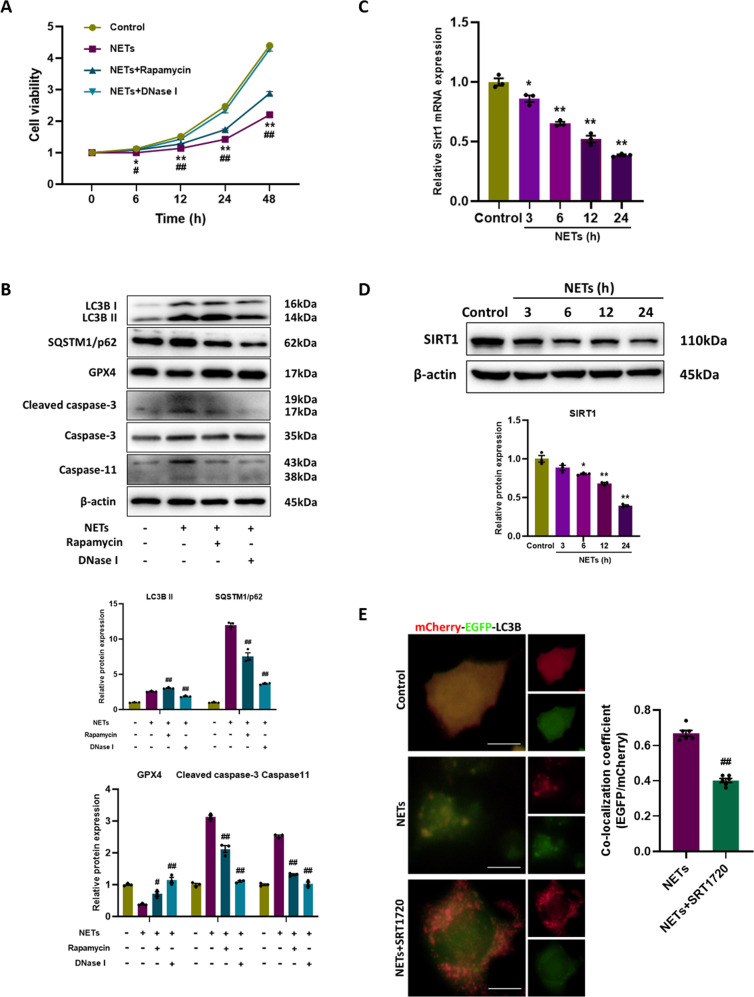
Activation of autophagic flux restores cell viability. **A** The cell viability of alveolar epithelial cells with different treatments. **B** Western blot images of autophagy signaling and cell death markers expression in alveolar epithelial cells with different treatments. **C** The mRNA level of Sirt1 in alveolar epithelial cells. **D** Western blot images of SIRT1 expression in alveolar epithelial cells. **E** Representative fluorescent images of alveolar epithelial cells transfected with mCherry-EGFP-LC3B and treated with NETs or NETs+SRT1720. Scale bar: 10 µm. Each bar shows means ± SEM. The data comparison between two groups was analyzed based on unpaired *t*-test. *p < 0.05, **p < 0.01 versus the control group. ^#^*p* < 0.05, ^##^*p* < 0.01 versus the NETs group.

To further verify the effects of NETs on autophagy, murine alveolar epithelial cells were treated with NETs, resulting in a significant decrease in cell viability (Fig. 4A). Moreover, by electron microscopy, we found that autophagic vesicles increased after NETs treatment (Fig. 4B), accompanied by increased LC3B staining in immunofluorescence images (Fig. 4C). NETs treatment induced increased expression of LC3B and SQSTM/p62, decreased expression of glutathione peroxidase 4 (GPX4) (a ferroptosis indicator), and increased expression of cleaved caspase-3 (an apoptosis indicator) and caspase-11 (a pyroptosis indicator), in a time-dependent manner (Fig. 4D). It can be concluded from these results that NETs cause blocked autophagic flux and the occurrence of cell death. Additionally, a tandem mCherry-EGFP-LC3B construct was used to further investigate the impaired autophagic flux. In cells treated with Earle’s Balanced Salt Solution (EBSS) (serum starvation to induce autophagic flux), red-only puncta representing autolysosomes were mainly observed. However, in cells treated with NETs, there was a substantial increase in the number of green-red (yellow) puncta representing autophagosomes, indicating impaired autophagy (Fig. 4E). Next, we found that rapamycin partially mitigated the decline in cell viability caused by NETs (Fig. 5A), activated autophagy and reduced ferroptosis, pyroptosis and apoptosis (Fig. 5B). In summary, the data confirm that NETs lead to the impairment of autophagic flux, and the activation of autophagy would restore cell viability.

Sirtuin 1 (SIRT1), the mammalian homolog of yeast silent information regulator (Sir 2), occupies a privileged position in cells, controls major metabolic and physiological processes, and is essential for increasing autophagic flux [19, 20]. Considering that SIRT1 is a critical protective factor in the alleviation of SI-ALI [21, 22], we examined the expression of SIRT1 after NETs treatment and found time-dependent decreases in mRNA and protein levels (Fig. 5C, D). Subsequently, we activated SIRT1 using SRT1720 (a SIRT1 activator) and assessed autophagic flux by the mCherry-EGFP-LC3B fluorescence analysis. Following SRT1720 administration, NETs-treated cells showed less green-red (yellow) puncta, indicating that the autophagic flux inhibited by NETs could be restored.

### NETs stimulate the METTL3 mediated N6-methyladenosine (m^6^A) modification in alveolar epithelial cells

**Fig. 6:**
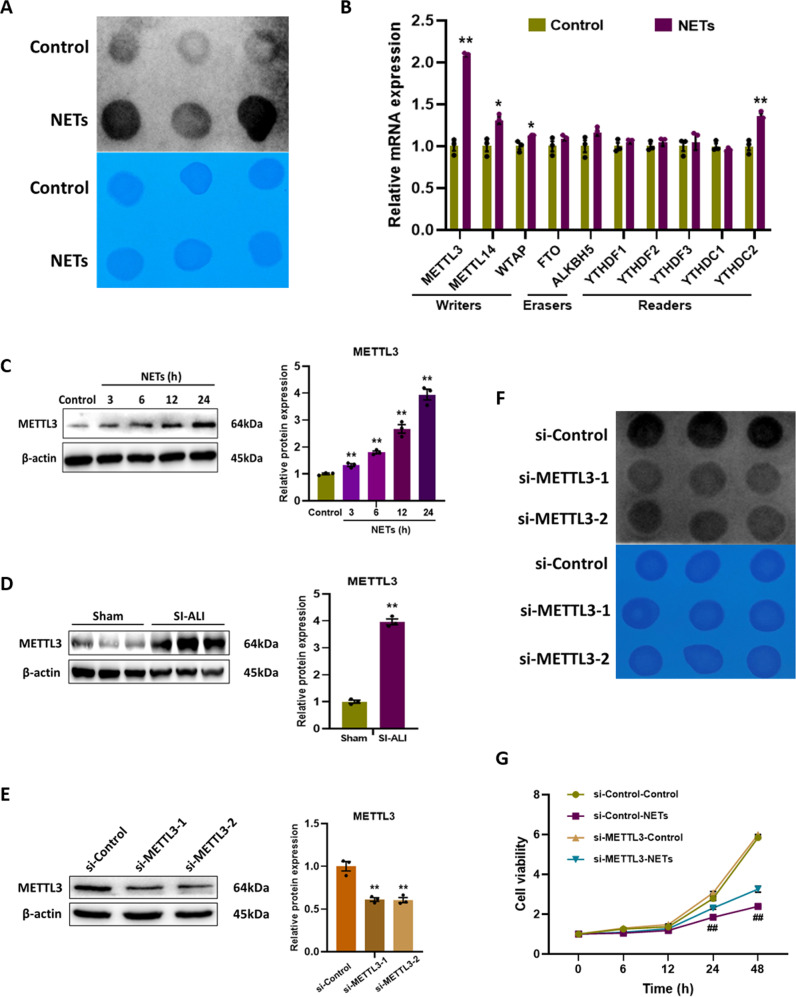
NETs stimulate the METTL3 mediated N6-methyladenosine (m^6^A) modification in alveolar epithelial cells. **A** Dot blot images of m^6^A levels in alveolar epithelial cells. **B** The mRNA levels of m^6^A modification related genes in alveolar epithelial cells. **C** Western blot images of METTL3 expression in alveolar epithelial cells. **D** Western blot images of METTL3 expression in lung tissues. **E** Western blot images of METTL3 expression in alveolar epithelial cells. **F** Dot blot images of m^6^A levels in alveolar epithelial cells. **G** The cell viability of alveolar epithelial cells with different treatments. Each bar shows means ± SEM. Data comparison between two groups was assessed by unpaired *t*-test. **p < 0.01 versus the control or sham or si-Control group. ^##^*p* < 0.01 versus the si-Control-NETs group.

Gene expression must be regulated finely at the transcriptional and translational levels in order to maintain cell viability. m^6^A methylation is the most common post-transcriptional modification of RNA in eukaryotes, and increasing evidence suggest that m^6^A methylation is crucial to many biological processes, including DNA damage, autophagy, and cell senescence [23–25]. Therefore, we investigated whether m^6^A methylation contributes to NETs-mediated cell damage. The overall m^6^A methylation level was detected by dot blot analysis, and it was significantly increased after NETs treatment (Fig. 6A). To identify the key m^6^A modification enzymes responsible for the elevated m^6^A methylation level, expression of m^6^A methyltransferase (METTL3, METTL14 and WTAP), m^6^A demethylases (FTO and ALKBH5) and m^6^A readers (YTHDF1-3, YTHDC1-2) were measured using RT-qPCR analysis. It was found that METTL3 expression was most markedly elevated (Fig. 6B), and western blot analysis confirmed that the protein expression level of METTL3 increased with time (Fig. 6C). Moreover, in vivo experiments verified that METTL3 expression was much higher in lung tissues of SI-ALI group than sham group (Figs. 6D, S3A).

Next, we transfected alveolar epithelial cells with si-Control or si-METTL3 and stimulated them with NETs, and found that the overall m^6^A methylation level decreased (Fig. 6F). The knockdown efficiency of METTL3 was validated by RT-qPCR and western blot analysis (Figs. 6E, S3B). As expected, knockdown of METTL3 attenuated the damage of NETs to alveolar epithelial cells, partially restoring cell viability (Fig. 6G). These findings above demonstrate that NETs-induced cell damage is dependent on the METTL3 mediated m^6^A modification.

### METTL3 knockdown activates autophagic flux and restores the mRNA stability of Sirt1

**Fig. 7:**
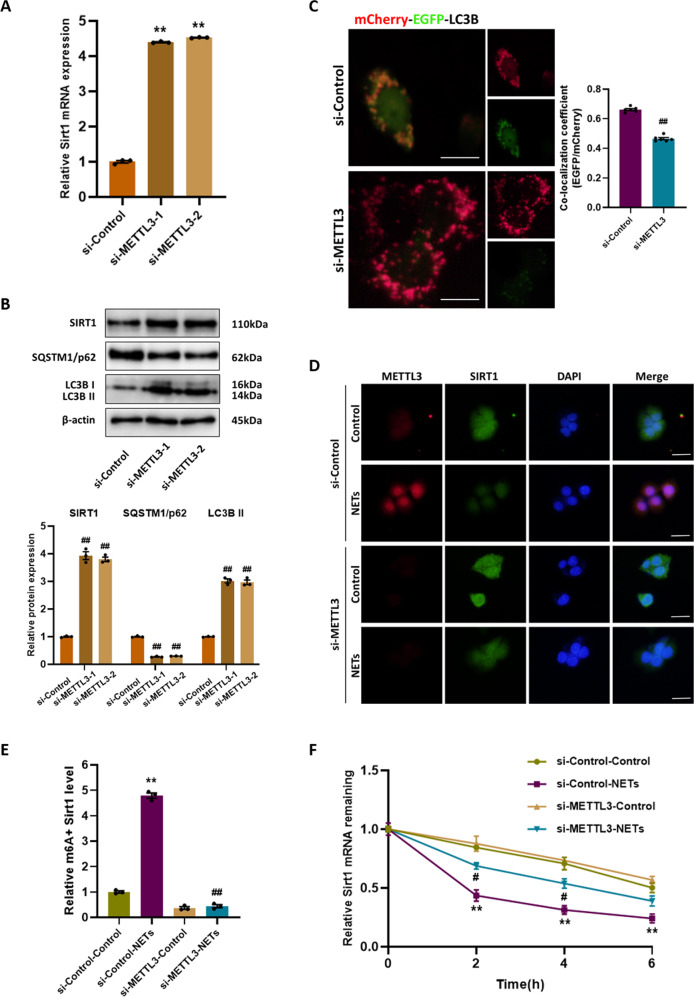
METTL3 knockdown activates autophagic flux and restores the mRNA stability of Sirt1. The alveolar epithelial cells were transfected with si-Control or si-METTL3 and treated with NETs. **A** The mRNA levels of Sirt1 expression in alveolar epithelial cells. **B** Western blot images of SIRT1 and autophagy signaling expression in alveolar epithelial cells. **C** Representative fluorescent images of alveolar epithelial cells transfected with mCherry-EGFP-LC3B and treated with NETs. Scale bar: 10 µm. **D** Representative images of immunofluorescence co-staining of METTL3 and SIRT1 in alveolar epithelial cells. Scale bar: 30 µm. **E** The methylated mRNA levels of Sirt1 in alveolar epithelial cells. **F** The stability of Sirt1 mRNA in alveolar epithelial cells. Each bar shows means ± SEM. The comparison between two groups was performed using unpaired *t*-test. **p < 0.01 versus the si-Control group. ^#^*p* < 0.05, ^##^*p* < 0.01 versus the si-Control-NETs group.

Based on the above results, we speculated that SIRT1 could be regulated by METTL3 mediated m^6^A modification, so we examined the effect of METTL3 on SIRT1 expression in alveolar epithelial cells exposed to NETs. SIRT1 mRNA and protein levels were restored by METTL3 knockdown as determined by RT-qPCR and western blotting (Fig. 7A, B). Furthermore, the protein level of SQSTM1/p62 decreased in si-METTL3 group (Fig. 7B), and mCherry-EGFP-LC3B fluorescence analysis showed more red-only puncta instead of green-red (yellow) puncta (Fig. 7C), indicating the recovery of autophagic flux. Immunofluorescence staining of SIRT1 and METTL3 further confirmed that the expression of SIRT1 inhibited by NETs was significantly upregulated with the knockdown of METTL3 (Fig. 7D). To investigate the mechanism of METTL3 downregulating SIRT1, we performed MeRIP-qPCR and found that m^6^A-specific antibody bound to Sirt1 mRNA more strongly after exposure to NETs. METTL3 knockdown markedly mitigated NETs-induced increase in methylated Sirt1 mRNA level (Fig. 7E). Next, actinomycin D was employed to examine the effect of m^6^A modification on Sirt1 mRNA stability. Upon NETs stimulation, Sirt1 mRNA degraded rapidly, which was suppressed after METTL3 knockdown (Fig. 7F), implying that METTL3 mediated m^6^A modification downregulated Sirt1 mRNA stability. Collectively, METTL3 mediated m^6^A modification is involved in the regulation of autophagy by affecting SIRT1 expression, according to these results.

## Discussion

**Fig. 8:**
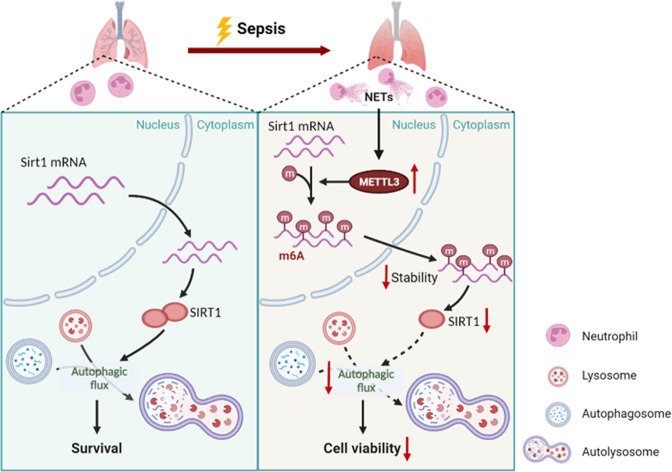
A proposed schematic illustrating the mechanism of damage to alveolar epithelial cells induced by NETs in sepsis. In the course of sepsis, NETs upregulate the expression of METTL3, resulting in increased Sirt1 mRNA m^6^A modification, thus reducing the stability of mRNA. Therefore, SIRT1 protein is decreased, which ultimately contributes to impaired autophagic flux and depressed cell viability.

In this study, we illuminated an important role of NETs in the pathogenesis of ALI/ARDS related to sepsis, and the destructive effect of NETs may be through blocking the autophagic flux of alveolar epithelial cells. Considering that SIRT1 can promote autophagic flux, we found that NETs activated the METTL3 mediated m^6^A modification, which resulted in decreased Sirt1 mRNA stability (Fig. 8).

Numerous studies have shown that NETs develop excessively in ALI/ARDS [26]. Firstly, there is a high toxicity associated with NETs to vascular endothelium and alveolar epithelial cells as a result of their contents of histones, MPO, and defensins, which act as danger-associated molecular patterns and trigger systemic inflammatory responses [27, 28]. Secondly, NETs contribute to immunothrombosis in ALI/ARDS and are positively correlated with disease severity [29]. As in our previous studies, immunothrombosis and disease progression were promoted by tissue factor-enriched NETs in SI-ALI [12]. In this study, we reconfirmed the deleterious effects of NETs, and inhibiting NETs alleviated systemic and pulmonary inflammation. In addition, we further demonstrated that these effects may be mediated by autophagy, which is reported for the first time. With the innovation of detection techniques, our results suggest that the assessment of the NETs level can be used as a novel biomarker for the diagnosis of ALI/ARDS. NETs may have diagnostic value in sepsis, but more research is needed.

Autophagy is mostly considered as a protective mechanism of cells to limit their injury under physiological responses or mild stress, as it is a lysosome-dependent process of removing damaged organelles and other cytoplasmic contents to lysosomes for degradation [30]. However, under severe or chronic stress, self-degradation or accumulation of harmful substances would result from excessive or insufficient autophagy, eventually leading to cell death [31]. Studies have shown that autophagy is triggered at the initial stage of sepsis development, but autophagic flux decreases with the progression of sepsis [32]. Moreover, in the mild sepsis model, the increase of autophagy was proportional to the severity of the challenge, while a proportional decrease in autophagy was observed during severe sepsis [33]. In our study, autophagy vesicles and LC3B expression were evidently increased after CLP in vivo or NETs treatment in vitro. However, further experiments showed that the autophagic flux was impaired. By intervening with rapamycin, lung injury and cell viability were somewhat improved, suggesting that keeping autophagy unobstructed was protective. These findings are similar to previous studies [34–36]. We hypothesized that autophagy activation caused by NETs irritation might serve as a protective mechanism, while blocked autophagic flux was an inevitable process of NETs resulting in cytotoxicity, which made cells unable to degrade damaged proteins and organelles in time, ultimately leading to declined cell viability. These findings suggest that NETs could affect the pathological prognosis by regulating autophagy, which adds new understanding to the role of NETs in the pathogenesis of ALI/ARDS, and provides theoretical basis for targeted-NETs therapy.

The m^6^A methylation is the most prevalent and conserved mRNA modification within eukaryotes, and is essential for a wide range of biological processes [37]. Nevertheless, few studies address the role of m^6^A in ALI/ARDS. Our study showed that m^6^A modification was increased in NETs-treated alveolar epithelial cells and METTL3 played a major role. METTL3 knockdown enhanced autophagic flux and protected NETs-treated alveolar epithelial cells to some extent. Furthermore, SIRT1 participates in the regulation of metabolism, autophagy and cell survival [19, 38]. It has also been reported that SIRT1 contributed to sepsis by regulating autophagy [39]. However, it is not yet clear what effect m^6^A modification has on Sirt1 mRNA. Mechanically, we proved that METTL3 methylated Sirt1 mRNA, reducing its stability and thus inhibiting protein expression.

In conclusion, we identified that the degradation of Sirt1 mRNA was epigenetically promoted by METTL3 mediated m^6^A modification in NETs-treated alveolar epithelial cells, and the autophagic flux was thus blocked. Our studies underscored the significance of NETs in the pathogenesis of sepsis-associated ALI/ARDS, and supported the development of therapeutic strategies targeting NETs.

## Materials and methods

### Human subjects

All human studies were approved by the Ethics Committee of Shanghai Cancer center, Fudan University (Protocol license number: 20180109-04), and performed according to the principles of the Declaration of Helsinki. The study included patients admitted to the ICU between January 2018 and December 2018 who signed a written informed consent form. ARDS was diagnosed on the basis of the 2012 Berlin criteria [5]: (1) acute onset of dyspnea with a history of aspiration within one week and incubation for mechanical ventilation support; (2) PaO_2_/FiO_2_ equal to or less than 300 mmHg over 48 h; (3) chest imaging shows new infiltration shadow. Exclusion criteria included: a history of cardiopulmonary arrest prior to admission to the ICU; history of connective tissue diseases such as vasculitis; pregnancy and vascular embolism.

### CLP mice model and treatments

Six- to eight-week-old male C57BL/6 J mice weighing 25–30 g were purchased from Shanghai Laboratory Animal Research Center (Shanghai, China). Our study was conducted in accordance with the Regulations for the Administration of Affairs Concerning Experimental Animals, and we obtained approval from the animal review committee of Zhongshan Hospital, Fudan University (Protocol license number: 2020-119). After random grouping, the CLP mice model was developed through the cecal ligation and puncture operation, based on previous research [12]. Briefly, the abdominal cavity was opened following intraperitoneal anesthesia with 1% pentobarbital sodium (1 mg/kg). The cecum was carefully separated and ligated with a 5–0 suture, and punctured using a 20-gauge needle. Then, we squeezed a small amount of feces before repositioning the cecum and closing the abdominal cavity. For rehydration, each animal was given 0.5 ml/10 g of normal saline. The sham group received the same surgery, but with no cecal ligation or puncture. If the mice needed treatment, following reagents were injected intraperitoneally: GSK484 (20 mg/kg, Cayman Chemical, Ann Arbor, MI, USA), Anti-Ly6G antibody (500 µg/mouse, ab238132, Abcam, Cambridge, MA, USA), DNase I (5 mg/kg, Sigma-Aldrich, St Louis, MO, USA), Rapamycin (5 mg/kg, MCE, Shanghai, China). At the end of the study, mice were euthanized, BALF, blood, and tissue were collected under sterile conditions.

### Histopathological analysis

Paraffin-embedded tissue sections were stained with haematoxylin and eosin (H&E). As described previously, the severity of ALI was assessed by pathologists blinded to the experimental information using a semiquantitative histology scoring method [10]. Briefly, the histological index of lung injury included haemorrhage, alveolar oedema, thickening of the alveolar septa and leukocyte infiltration. From 0 to 3 (0 = normal; 1 = mild; 2 = moderate; 3 = severe), we graded each indicator, and then calculated the total lung injury score.

### Isolation of neutrophils and NETs production

Neutrophils were isolated from human or mouse blood according to the kit’s instructions. Briefly, the blood samples were layered over neutrophil separation medium and centrifuged at 800 g for 30 min at room temperature. The lower leukocyte band containing neutrophils was collected, followed by schistocyte and washing with PBS. The obtained cells were resuspended in DMEM (Gibco, US) supplemented with 10% fetal bovine serum (FBS; Gibco). Isolated neutrophils were treated with 50 nM PMA (MKBio, Shanghai, China) for four hours. After removing the supernatant, NETs adhered at the bottom were washed down by pipetting 2 ml of cell culture medium and were centrifuged to remove cell debris.

### Quantification of cf-DNA and MPO-DNA complexes

The cf-DNA and MPO-DNA complexes concentration were measured using the Quant-iT™ PicoGreen kit (Invitrogen, MA, USA) and ELISA kit (ab119605, Abcam) according to the manufacturer’s instructions respectively.

### Immunofluorescence

Paraffin-embedded tissue sections were deparaffinized, rehydrated, and antigen-retrieved, and cells were fixed and permeabilized. After blocking, they were incubated with antibodies against CitH3 (1:100, ab5103, Abcam), MPO (1:50, AF3667, R&D Systems, MN, USA), Ly6G (1:100, 127601, Biolegend, CA, USA), LC3B (1:100, ab192890, Abcam), LAMP1 (1:100, ab208943, Abcam), METTL3 (1:100, ab195352, Abcam), SIRT1 (1:100, ab110304, Abcam) and fluorescent secondary antibodies. Finally, the nuclei were stained with DAPI. The slices were viewed under an Olympus microscope (Tokyo, Japan).

### Immunohistochemistry

Paraffin-embedded tissue sections were deparaffinized, rehydrated, treated with 0.3% hydrogen peroxide, and processed with heat induction for antigen retrieval. The sections were subsequently incubated with primary antibodies LC3B (1:50), METTL3 (1:50) overnight at 4 °C. After using anti-rabbit HRP antibody, diaminobenzidine staining, and counterstaining with hematoxylin, positive staining was revealed. Slides were examined under light microscopy (Carl Zeiss, Jena, Germany).

### Lung wet-to-dry ratio

After absorbing surface water of the right lung tissue and obtaining its wet weight, each specimen was dried at 70 °C for 48 h. The wet weight/dry weight ratio was calculated by dividing the wet weight by the dry weight.

### Quantification of inflammatory indicators

IL-1β, IL-6, and TNF-α levels in the mouse plasma and BALF were measured by using IL-1β ELISA kit (abs520001, Absin, Shanghai, China), IL-6 High Sensitivity ELISA kit (abs552805, Absin), and TNF-α ELISA kit (abs520010, Absin).

### Transmission electron microscopy (TEM)

Fixation in 2.5% glutaraldehyde, postfixation with 1% osmic acid, dehydration with graded ethanol, and embedding in 812 resin were followed by thin section staining with 2% uranyl acetate. Pictures were taken with a Hitachi HT7700 microscope (Tokyo, Japan).

### Cell culture and treatments

Murine Lung Epithelial-12 (MLE12) was obtained from the American Type Culture Collection (ATCC, Manassas VA, USA) and cultured in DMEM/F12 (Gibco) supplemented with 10% FBS at 37 °C, 5% CO_2_ humidified incubator. EBSS was purchased from BasalMedia (Shanghai, China). SRT1720 was purchased from Selleck Chemicals (Shanghai, China). By using lipofectamine 3000 (Invitrogen), MLE12 were transfected with METTL3 small interfering RNA (si-METTL3) or negative small interfering RNA (si-Control) for interference treatment. The primer sequences of si-METTL3 were listed in Supplementary Table 2.

### Cell viability assay

A Cell-Counting Kit 8 (Dojindo Corp., Kumamoto, Japan) was used to test relative cell viability according to the manufacturer’s instructions.

### The mCherry-EGFP-LC3B fluorescence microscopy assay

Cells were seeded in 24-well plates (5 × 10^4^ cells/well) one day before transfection. Cells were transfected with AdM-CMV-mCherry-EGFP-LC3B adenovirus (MOI = 100, Vigene, Jinan, China) for 24 h and then subjected to different treatments. Images were taken using an Olympus microscope. Quantification of yellow puncta and red-only puncta was measured using the EGFP/mCherry colocalization efficiency.

### m^6^A dot blot

TRIzol method was used to extract the total RNA. Dynabeads mRNA Purification Kit (Thermo Fisher, MA, USA) was used to isolate the purified mRNA, which was denatured and dotted on Amersham Hybond-N+ membranes, then cross-linked for 10 min using UVP. Finally, m^6^A levels were measured with the anti-m^6^A antibody (1:1000, 202003, Synaptic Systems, Goettingen, Germany).

### m^6^A-RNA immunoprecipitation and MeRIP-qPCR

Based on previous reports, the m^6^A-RNA immunoprecipitation was performed [40, 41]. The antibodies were anti-m^6^A and control IgG (2729 S, Cell Signaling Technology, Danvers, MA, USA). The qPCR assay was conducted and the m^6^A-RIP fraction normalized to the input was calculated.

### Assessment of Sirt1 mRNA stability

RNA extraction and RT-qPCR were performed following treatment with 5 μg/ml actinomycin D (Selleck Chemicals) for 0, 2, 4 and 6 h.

### Quantitative Real-Time PCR

The total RNA was extracted using TRIzol reagent (Thermo Fisher), and reverse-transcribed into cDNA using PrimeScript RT reagent kit (RR036A, Takara, Shinga, Japan). RT-qPCR was performed with a TB Green PCR kit (RR820A, Takara) and Bio-Rad system with three repetitions per well. Quantification of gene expression was normalized to endogenous β-actin expression. The primer sequences were listed in Supplementary Table 3.

### Western Blot

Cells or tissues were lysed in RIPA Buffer (Solarbio, Beijing, China) containing proteinase inhibitor cocktail. Proteins were separated with sodium dodecyl sulfate-polyacrylamide gel electrophoresis (SDS-PAGE) and transferred onto polyvinylidene fluoride (PVDF) membranes. The membranes were blocked and incubated with primary antibodies including CitH3 (1:1000), MPO (1:1000), LC3B (1:1000), SQSTM1/p62 (1:1000), GPX4 (1:2000, ab125066, Abcam), cleaved caspase-3 (1:1000, 9661, Cell Signaling Technology), caspase-3 (1:1000, 9662, Cell Signaling Technology), caspase-11 (1:1000, 14340, Cell Signaling Technology), SIRT1 (1:1000), METTL3 (1:1000), β-Actin (1:3000, 3700, Cell Signaling Technology). Signals were detected with a ECL chemiluminescence kit (Tanon, Shanghai, China) after HRP-conjugated secondary antibodies incubation.

### Statistical analysis

All statistical analyses were carried out using SPSS 23.0 and GraphPad Prism 8 software. Data were expressed as means ± standard error of the means (SEM). All experiments were repeated at least three times independently, and two-tailed unpaired t-test was applied for two groups comparison analysis. *P* < 0.05 was considered statistically significant (*/^#^*P* < 0.05, **/^##^*P* < 0.01).

## Supplementary information

Supplementary Table

Supplementary figure legends

Supplementary Figure 1

Supplementary Figure 2

Supplementary Figure 3

Original Data File

## Data Availability

All relevant data are included in this published article and its supplementary files.
