# Supplementary material for: Neutrophil extracellular traps-triggered impaired autophagic flux via METTL3 underlies sepsis-associated acute lung injury
Source: Cell Death Discov. 2022 Aug 27;8:375. doi: 10.1038/s41420-022-01166-3 (PMC9420153; doi:10.1038/s41420-022-01166-3)
Supplement: Supplementary file 1 — Supplementary Table [file 41420_2022_1166_MOESM1_ESM.docx]

**Supplementary Table 1. Baseline characteristics of healthy controls (HC) and ARDS patients.**

|  | Healthy Control (N = 25) | ARDS (N=24) | P value |
| --- | --- | --- | --- |
| Gender (n, %) |  |  | 0.879 |
| Male | 13 (52.0%) | 13 (54.2%) |  |
| Female | 12 (48.0%) | 11 (45.8%) |  |
| Ages (years) | 43.8±12.9 | 46.2±13.4 | 0.526 |
| BMI (kg/m^2^) | 27.8±5.5 | 28.6±4.2 | 0.571 |
| Neutrophils (10^9^/L) | 3.6±0.7 | 7.6±2.9 | <0.05 |
| Monocytes (10^9^/L) | 0.5±0.3 | 0.7±0.4 | 0.053 |
| Lymphocytes (10^9^/L) | 2.0±0.4 | 1.8±0.5 | 0.128 |
| Platelets (10^9^/L) | 247.5±43.8 | 384.8±169.5 | <0.05 |
| Hemoglobin (g/L) | 136.2±14.1 | 97.5±19.4 | <0.05 |
| Albumin (g/L) | 47.9±3.5 | 29.2±5.6 | <0.05 |
| Plasma cytokine level (pg/ml) |  |  |  |
| IL-6 | 34 (30-39) | 815 (572-1103) | <0.05 |
| TNF-α | 52 (45-61) | 139 (82-166) | <0.05 |
| IL-1β | 22 (18-31) | 71 (54-93) | <0.05 |
| PaO_2_/FiO_2_ (mmHg) | 443±34 | 214±36 | <0.05 |

Data are expressed as n (%), mean ± SD or median (interquartile range [IQR]). BMI: body mass index.

**Supplementary Table 2. Primer sequence of si-METTL3.**

|  | Sense | Antisense |
| --- | --- | --- |
| si-METTL3-1 | GACGAAUUAUCAAUAAGCACA | UGCUUAUUGAUAAUUCGUCUG |
| si-METTL3-2 | GCAAAUAUGUUCACUAUGAAA | UCAUAGUGAACAUAUUUGCAG |

**Supplementary Table 3. Primer sequences for RT-qPCR.**

| Gene | Forward | Reverse |
| --- | --- | --- |
| SIRT1 | TGATTGGCACCGATCCTCG | CCACAGCGTCATATCATCCAG |
| METTL3 | CTGGGCACTTGGATTTAAGGAA | TGAGAGGTGGTGTAGCAACTT |
| METTL14 | GAGCTGAGAGTGCGGATAGC | GCAGATGTATCATAGGAAGCCC |
| WTAP | ATGGCACGGGATGAGTTAATTC | TTCCCTTAAACCAGTCACATCG |
| FTO | GACACTTGGCTTCCTTACCTG | CTCACCACGTCCCGAAACAA |
| ALKBH5 | GCGCGGTCATCAACGACTA | ATCAGCAGCATACCCACTGAG |
| YTHDF1 | ACAGTTACCCCTCGATGAGTG | GGTAGTGAGATACGGGATGGGA |
| YTHDF2 | GAGCAGAGACCAAAAGGTCAAG | CTGTGGGCTCAAGTAAGGTTC |
| YTHDF3 | GATCAGCCTATGCCATATCTGAC | CCCCTGGTTGACTAAAAACACC |
| YTHDC1 | GAGAATGGAGTCTACTGACACCA | ACAGACGAATTTTTCGATCAGCA |
| YTHDC2 | GAAGATCGCCGTCAACATCG | GCTCTTTCCGTACTGGTCAAA |
| β-actin | GTGACGTTGACATCCGTAAAGA | GCCGGACTCATCGTACTCC |
