# Supplementary material for: Neutrophil extracellular traps-triggered impaired autophagic flux via METTL3 underlies sepsis-associated acute lung injury
Source: Cell Death Discov. 2022 Aug 27;8:375. doi: 10.1038/s41420-022-01166-3 (PMC9420153; doi:10.1038/s41420-022-01166-3)
Supplement: Supplementary file 2 — Supplementary figure legends [file 41420_2022_1166_MOESM2_ESM.docx]

# Supplementary Figure Legends

# Supplementary Figure 1. CLP was used to construct mice models of SI-ALI.

**(A)** H&E staining of the lung tissues. Scale bar: 50 µm. **(B)** Semiquantitative histological scores of lung injury in groups (n = 6). The error bar shows means

± SEM. Data comparison between two groups was performed using unpaired t-test. ** p < 0.01 versus the sham group.

# Supplementary Figure 2. Autophagy activation ameliorates inflammatory in sepsis mice.

**(A)** H&E staining of the lung tissues. Scale bar: 50 µm. **(B)** Semiquantitative histological scores of lung injury in groups (n = 6). **(C)** The lung wet/dry ratio (n = 6). **(D)** The levels of TNF-α, IL-1β, and IL-6 in plasma (n = 6). **(E)** The

levels of TNF-α, IL-1β, and IL-6 in BALF (n = 6). Each bar shows means ± SEM. The comparison between two groups was analyzed by unpaired t-test. ** p < 0.01 versus the sham group; ## p < 0.01 versus the saline group.

# Supplementary Figure 3. METTL3 mediated m6A modification is involved in NETs-induced cell damage.

**(A)** Representative images of immunohistochemical staining for METTL3 in lung tissues. Scale bar: 50 µm. **(B)** The mRNA levels of Mettl3 expression in alveolar epithelial cells. Each bar shows means ± SEM. The comparison between two groups was conducted by unpaired t-test. ** p < 0.01 versus the si- Control group.
