# Supplementary figures and images for: Neutrophil extracellular traps-triggered impaired autophagic flux via METTL3 underlies sepsis-associated acute lung injury
Source: Cell Death Discov. 2022 Aug 27;8:375. doi: 10.1038/s41420-022-01166-3 (PMC9420153; doi:10.1038/s41420-022-01166-3)

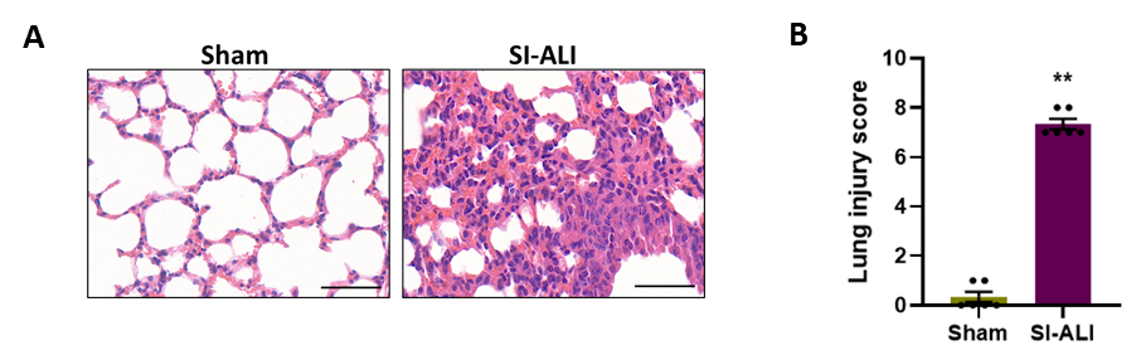

Supplement: Supplementary file 3 — Supplementary Figure 1 [file 41420_2022_1166_MOESM3_ESM.png]

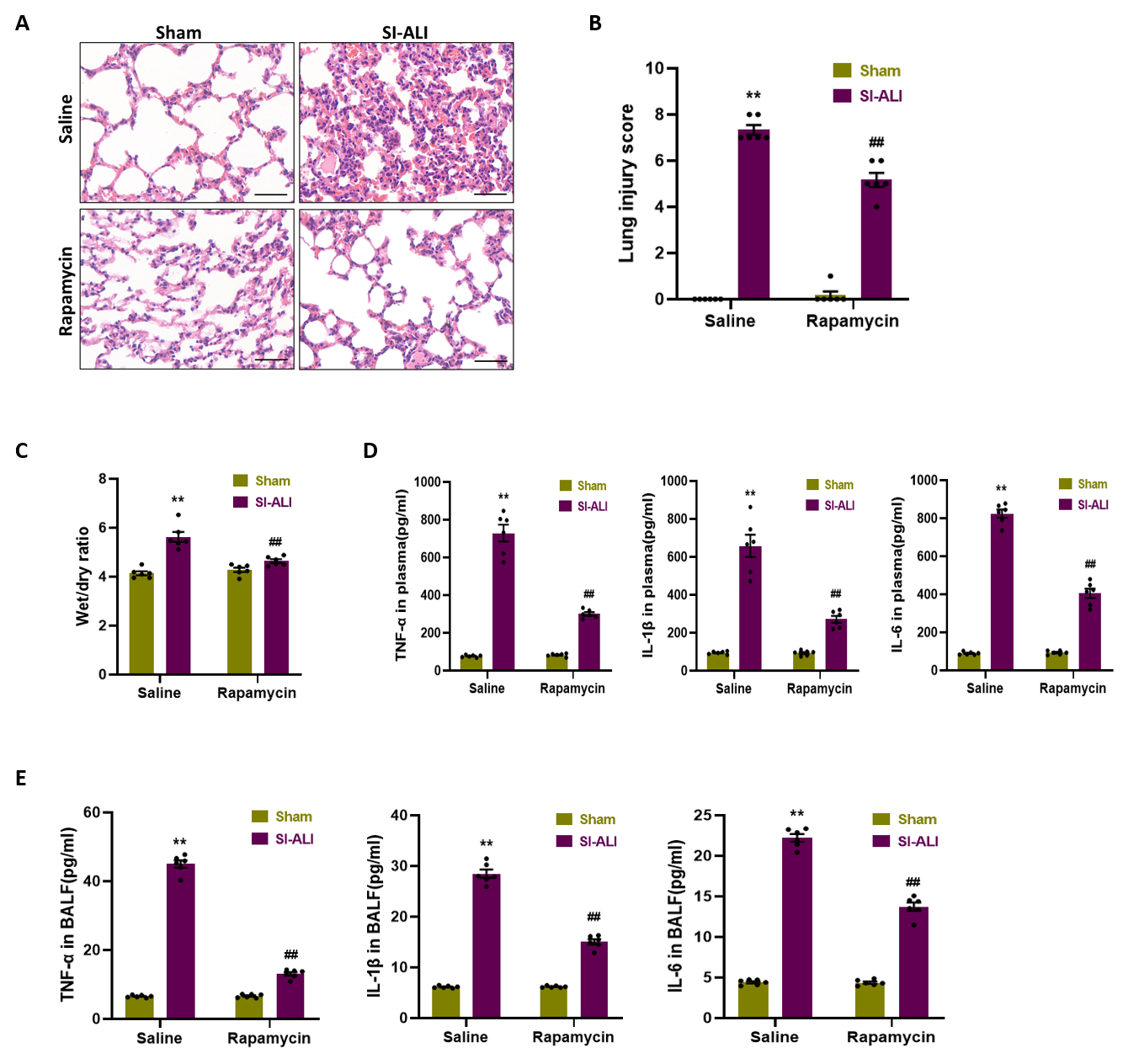

Supplement: Supplementary file 4 — Supplementary Figure 2 [file 41420_2022_1166_MOESM4_ESM.png]

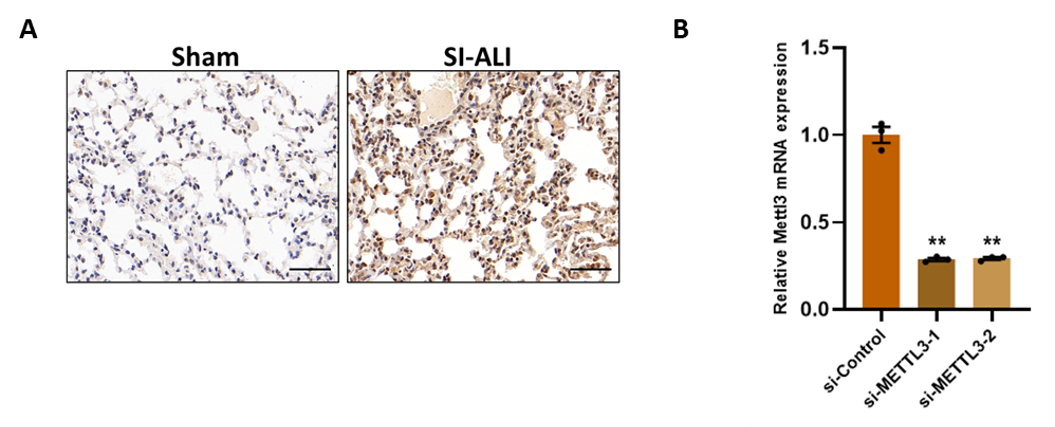

Supplement: Supplementary file 5 — Supplementary Figure 3 [file 41420_2022_1166_MOESM5_ESM.png]
