# Supplementary material for: Neutrophil extracellular traps-triggered impaired autophagic flux via METTL3 underlies sepsis-associated acute lung injury
Source: Cell Death Discov. 2022 Aug 27;8:375. doi: 10.1038/s41420-022-01166-3 (PMC9420153; doi:10.1038/s41420-022-01166-3)

Figure 1.G

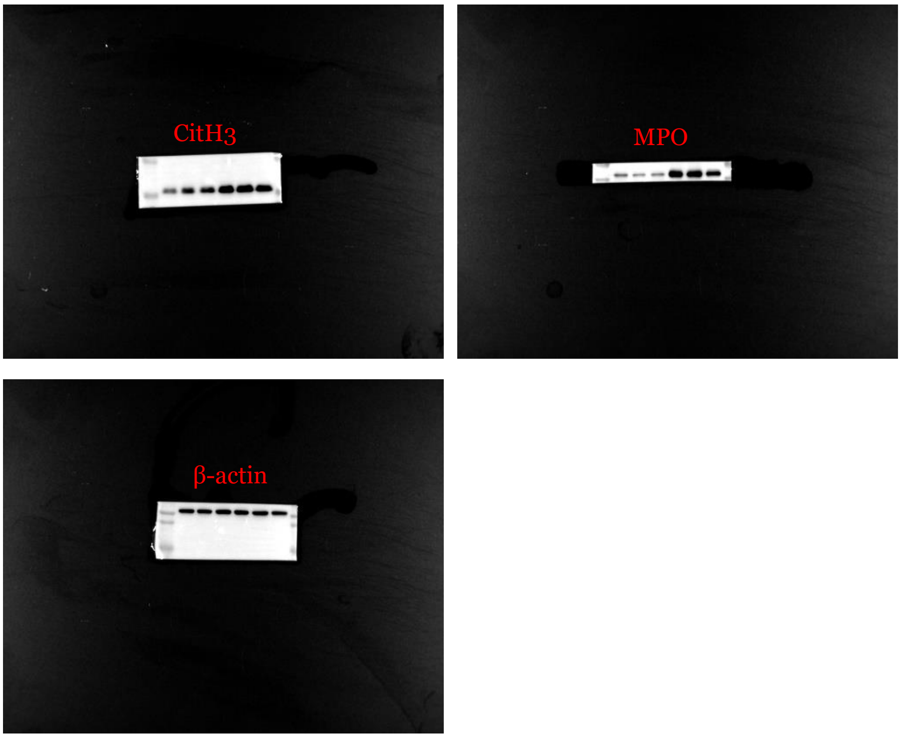

Figure 3.C

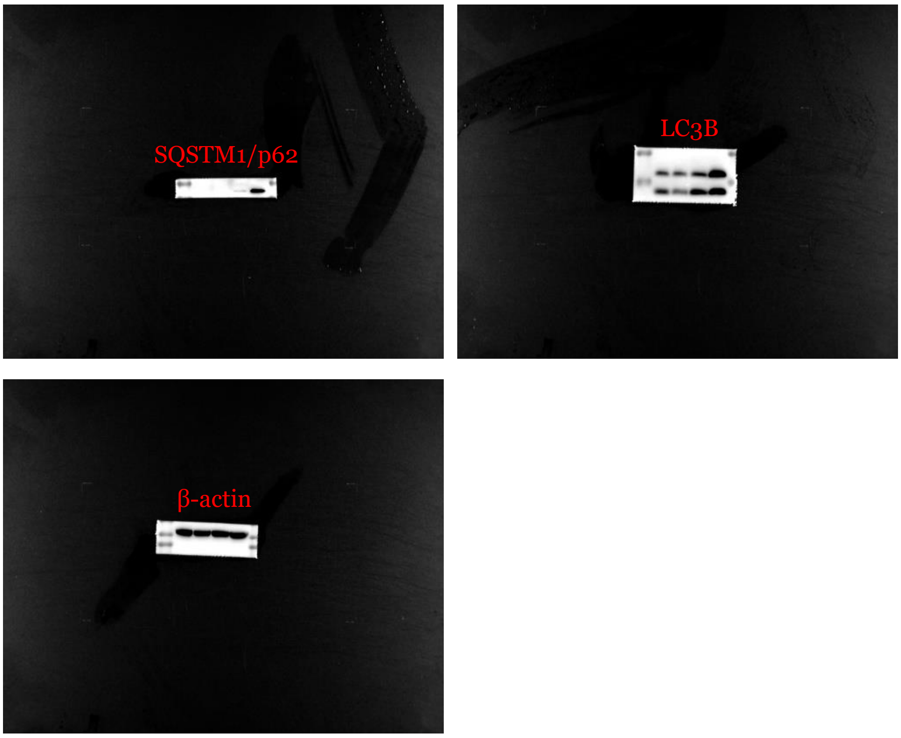

Figure 3.F

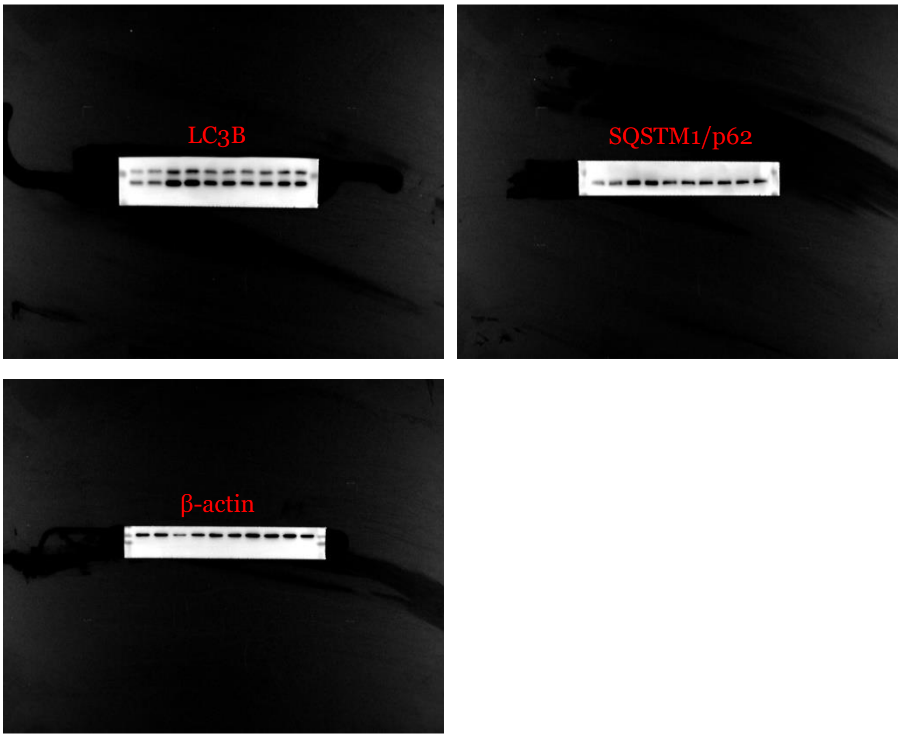

Figure 4.D

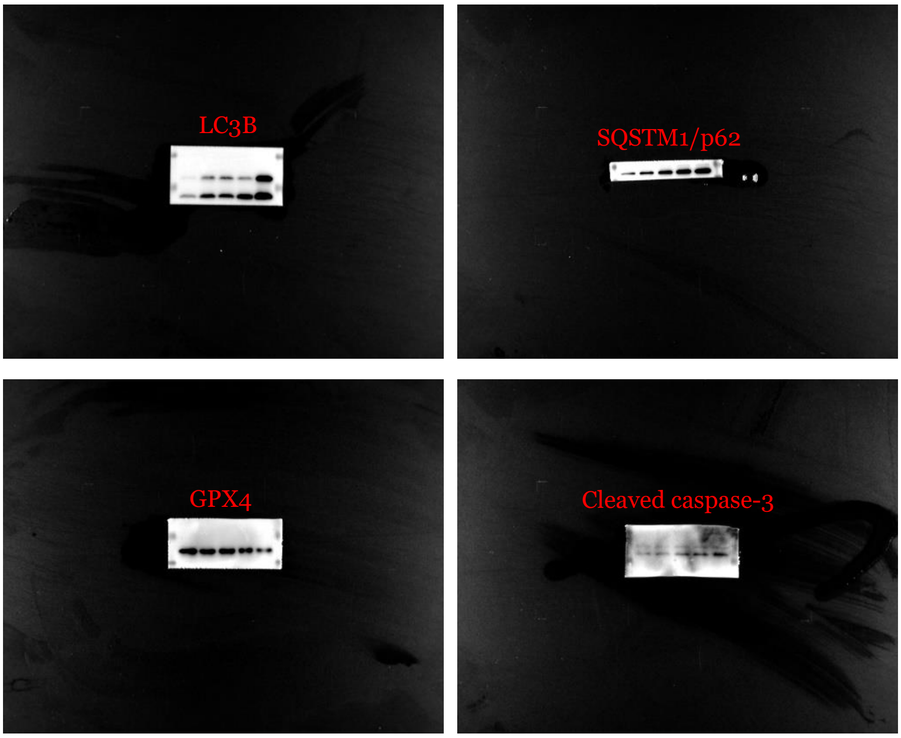

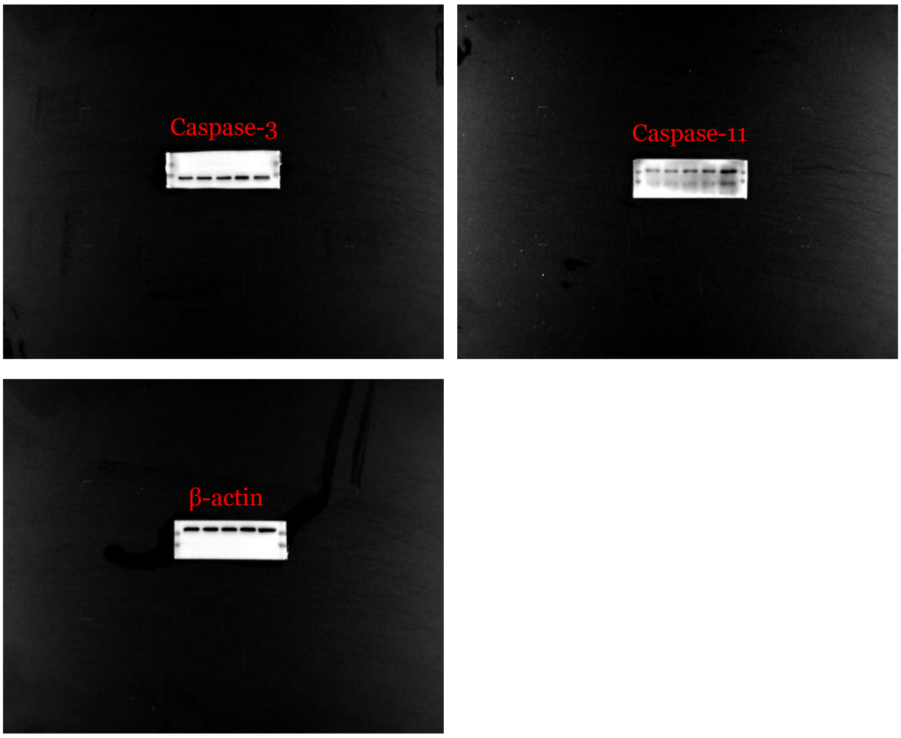
Caspase-3
Caspase-11
β-actin

Figure 5.B

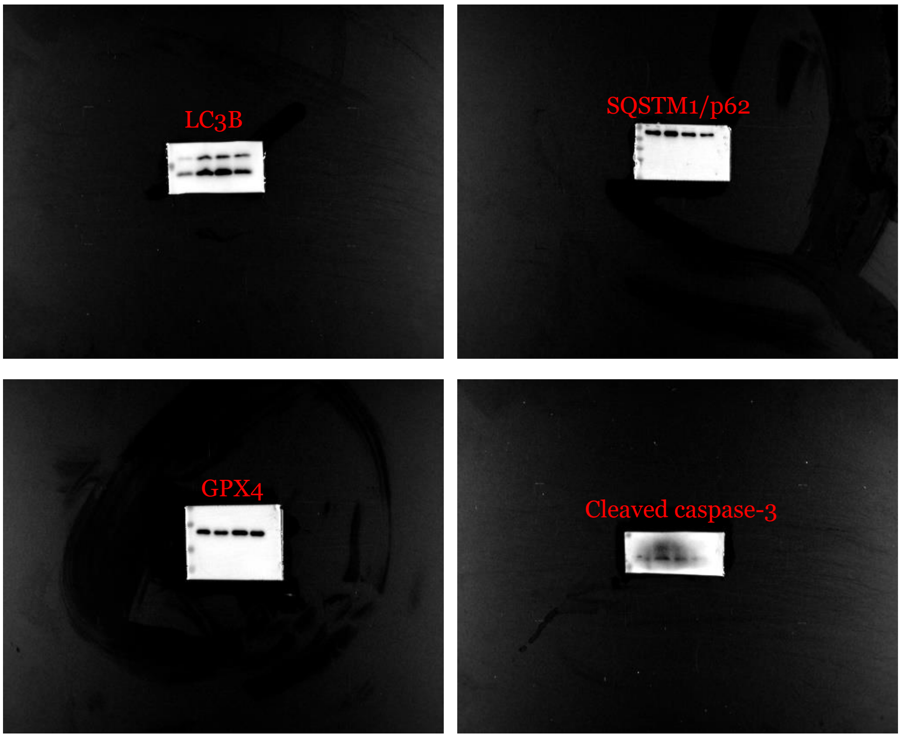
LC3B
SQSTM1/p62
GPX4
Cleaved caspase-3

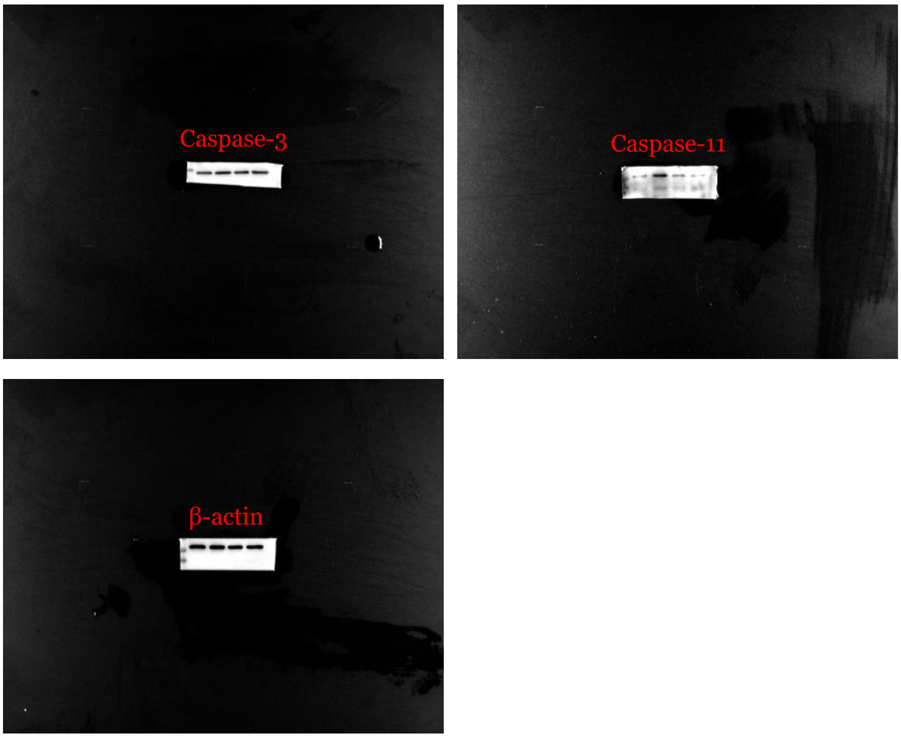

Figure 5.D

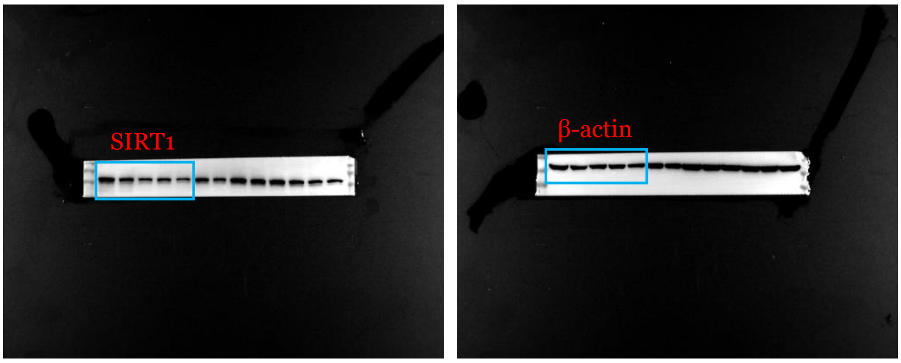

Figure 6.A

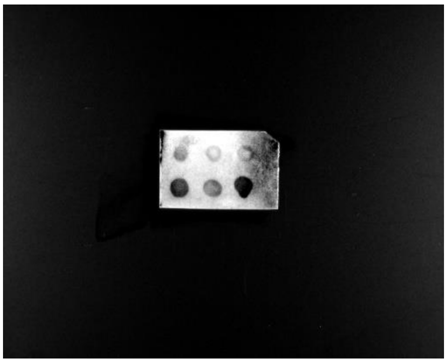

Figure 6.C

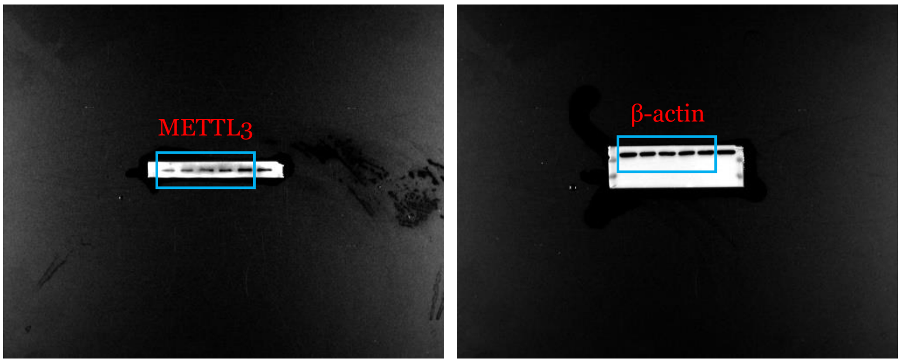

Figure 6.D

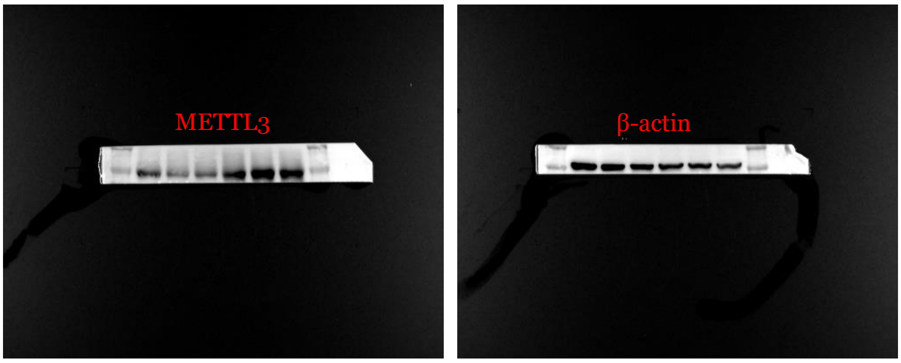

Figure 6.E

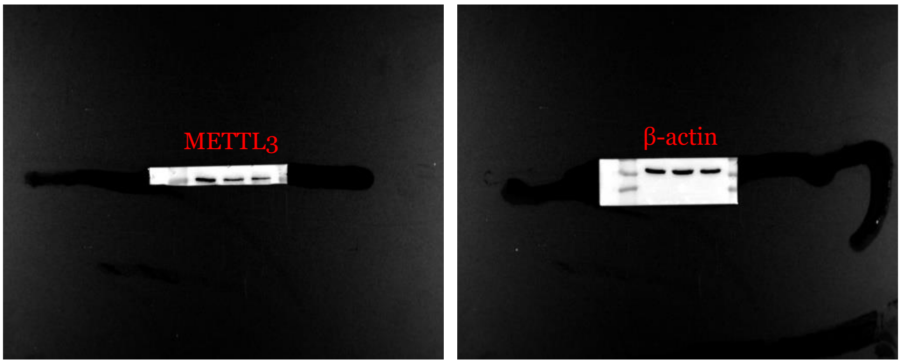

Figure 6.F

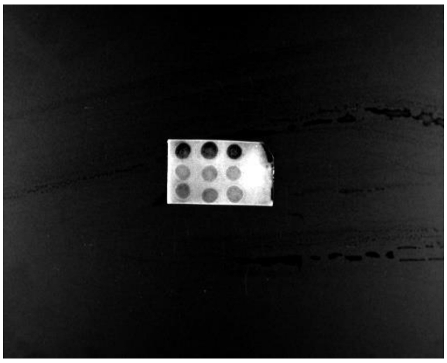

Figure 7.B

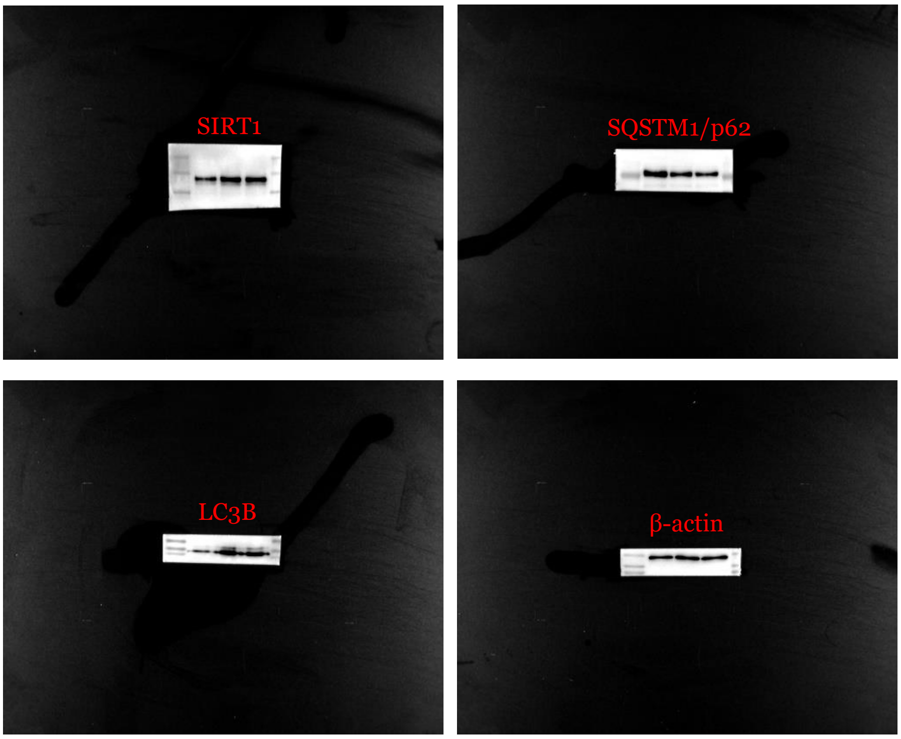

Supplement: Supplementary file 6 — Original Data File [file 41420_2022_1166_MOESM6_ESM.pdf]
